# Usefulness and Limits of Tractography for Surgery in the Precentral Gyrus—A Case Report

**DOI:** 10.3390/clinpract12020027

**Published:** 2022-04-11

**Authors:** Tim Wende, Florian Wilhelmy, Johannes Kasper, Gordian Prasse, Christian Franke, Felix Arlt, Clara Frydrychowicz, Jürgen Meixensberger, Ulf Nestler

**Affiliations:** 1 Department of Neurosurgery, University Hospital Leipzig, Liebigstr. 20, 04103 Leipzig, Germany; florian.wilhelmy@medizin.uni-leipzig.de (F.W.); johannes.kasper@medizin.uni-leipzig.de (J.K.); christian.franke@medizin.uni-leipzig.de (C.F.); felix.arlt@medizin.uni-leipzig.de (F.A.); juergen.meixensberger@medizin.uni-leipzig.de (J.M.); ulf.nestler@medizin.uni-leipzig.de (U.N.); 2 Institute of Neuroradiology, University Hospital Leipzig, 04103 Leipzig, Germany; gordian.prasse@medizin.uni-leipzig.de; 3 Institute of Neuropathology, University Hospital Leipzig, 04103 Leipzig, Germany; clara.frydrychowicz@medizin.uni-leipzig.de

**Keywords:** tractography, glioblastoma, precentral gyrus, transcranial magnetic stimulation, direct electrical stimulation

## Abstract

The resection of tumors within the primary motor cortex is a constant challenge. Although tractography may help in preoperative planning, it has limited application. While it can give valuable information on subcortical fibers, it is less accurate in the cortical layer of the brain. A 38-year-old patient presented with paresis of the right hand and focal epileptic seizures due to a tumor in the left precentral gyrus. Transcranial magnetic stimulation was not applicable due to seizures, so microsurgical resection was performed with preoperative tractography and intraoperative direct electrical stimulation. A histopathological assessment revealed a diagnosis of glioblastoma. Postoperative magnetic resonance imaging (MRI) showed complete resection. The paresis dissolved completely during follow-up. Surgery within the precentral gyrus is of high risk and requires multimodal functional planning. If interpreted with vigilance and consciousness of the underlying physical premises, tractography can provide helpful information within its limitations, which is especially subcortically. However, it may also help in the identification of functional cortex columns of the brain in the presence of a tumor.

## 1. Introduction

Operations within cerebral areas of primary function, where tissue damage will most likely lead to clinically relevant neurological deficits, present a constant challenge in the field of neurosurgery [1]. Especially for subcortical lesions, tractography is becoming a steadily improving tool for preoperative planning of microsurgical resection of intracerebral tumors [2]. However, there are limitations to this anatomical approach, so there are ongoing discussions on its applicability for tumor resection in high-risk cerebral locations such as the precentral gyrus [3].

The key advantage of tractography is the anatomical identification of subcortical nerve fibers. This has advanced neurosurgical planning and assists in risk stratification before surgery [4]. Therefore, on the other hand, tractography is not the first choice for preoperative assessment of cortical lesions. However, as the human cortex is organized in columns [5] and since the corticospinal tract is a descending pathway, leaving the cortex orthogonally [6], it stands to reason that tractography may indicate specific cortex regions at the terminals of the calculated streamlines. It has accordingly been shown that, in certain circumstances where other methods are not available due to different reasons, tractography may facilitate the identification of cortex areas with primary function [7].

This case report demonstrates how these findings and considerations may be useful for improving patient safety in high-risk surgery.

## 2. Case Presentation

A 38-year-old male patient presented to our department after first onset of focal seizures in the right hand. Magnetic resonance imaging (MRI) revealed a cystic tumor of 32 mm with bordering contrast enhancement in the left precentral gyrus. After the start of anticonvulsant treatment with levetiracetam by our department, no further seizures occurred. A mild weakness of the right hand slowly improved after the start of antiedematous treatment with dexamethasone. It may be of further interest that the son of the patient had succumbed to an anaplastic ependymoma at the age of four, which is an unusual coincidence and raises the question of an underlying cause, considering that ependymoma is sometimes classified as a kind of glioma [8].

Diffusion tensor imaging tractography was performed as described before (Brainlab AG, Munich, Germany) [9]. We acquired an anatomical T1-weighted MRI with contrast enhancement as well as a diffusion tensor imaging sequence with 32 directions. The latter was corrected for eddy currents and co-registered with the anatomical sequence. An atlas segmentation was applied and used for the placement of regions of interest. The fractional anisotropy cutoff was 0.15, minimum streamline length was 30 mm, and maximum streamline length was 250 mm. Brainstem and left internal capsule were selected for inclusion with a margin of 10 mm. We excluded both temporal lobes, the cerebellum, and the right internal capsule. No streamlines were excluded manually in this case.

**Figure 1:**
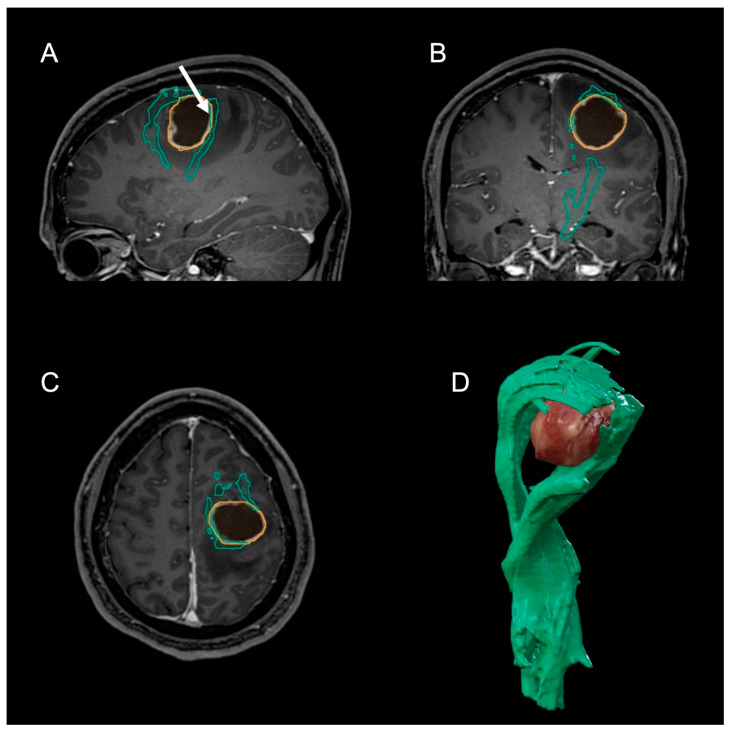
Preoperative MRI in T1-weighted sequence with contrast enhancement (**A**–**C**). Arrow: location of positive intraoperative DES at 2 mA. Note the cystic tumor (orange outlines) within the left precentral gyrus. Green outlines: CST. (**D**) Preoperative tractogram of the left CST (green) suggests fibers ventral and dorsal from the tumor. Intraoperative DES revealed primary motor neurons only dorsally. (MRI: magnetic resonance imaging; CST: corticospinal tract; DES: direct electrical stimulation).

The tumor was found to dissect the origin of the corticospinal tract (CST), and the tractogram suggested that the tumor lay beneath the primary motor cortex, with axons displaced ventrally (Figure 1). Preoperative verification with transcranial magnetic stimulation was not feasible due to the recent onset of epileptic seizures.

Therefore, we planned a microsurgical resection of the tumor using intraoperative electrophysiological monitoring and 5-aminolevulinic acid fluorescence. The patient gave his informed consent after weighing the risks and benefits with the assigned surgeon. Due to the partially inconclusive results of preoperative tractography, direct electrical stimulation (DES) at 15 mA was used during surgery, complemented by ultrasound guidance. This disclosed the functional location of the tumor 5 mm deep within the left precentral gyrus without provoking an epileptic fit. With regard to the delicate location of the tumor, the cyst was aspirated with a Cushing cannula and DES was then performed at 2 mA within the cyst. This revealed that primary motor neurons were only present dorsal to the cyst, so that a microsurgical resection of the tumor could be performed. Due to the excitability of the right hand by DES, some remnants of fluorescent tissue had to be left in situ.

**Figure 2:**
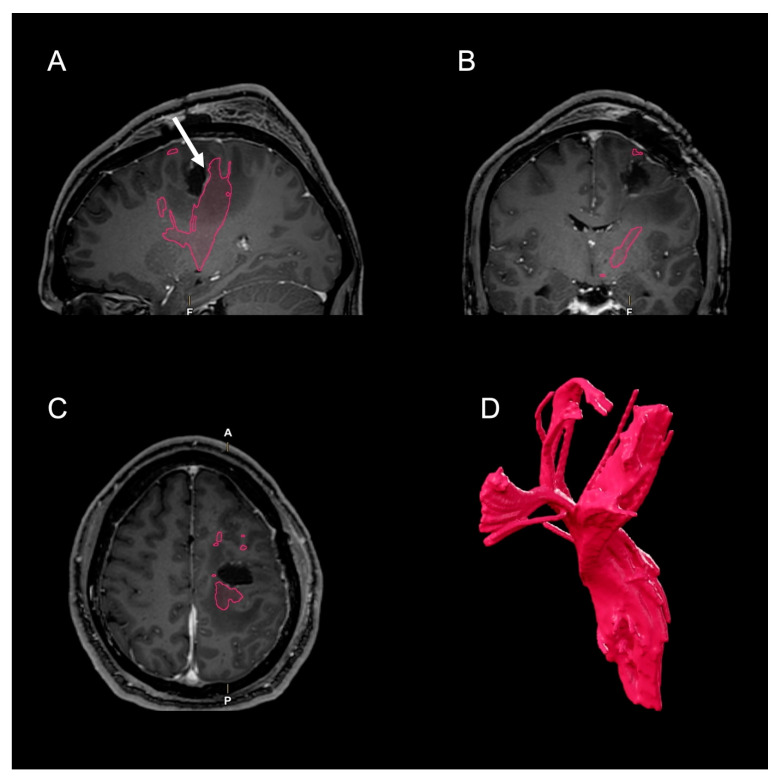
Postoperative MRI in T1-weighted sequence with contrast enhancement (**A**–**C**). Arrow: location of positive intraoperative DES at 2 mA. The contrast-enhancing parts of the tumor were completely resected. Edema was already reduced. Red outlines: CST. (**D**) Postoperative tractogram of the left CST (red) still shows some fibers ventral to the situs. However, the dorsal parts appear much stronger than preoperatively. (MRI: magnetic resonance imaging; CST: corticospinal tract; DES: direct electrical stimulation).

Postoperatively, there was no aggravation of the preexisting paresis, which completely resolved after two weeks. Postoperative MRI showed a complete resection of the cyst with its contrast enhancing capsula (Figure 2). The postoperative tractogram of the left CST shows less streamlines ventrally of the situs. In contrast, the dorsal parts appear much stronger than preoperatively.

Anticonvulsant medication was continued after surgery for prophylactic reasons.

Histopathological assessment and further molecular analysis of the specimen resulted in the diagnosis of glioblastoma WHO grade 4, isocitrate dehydrogenase wildtype, O(6)-methylguanine-methyltransferase promoter methylation 26.6%. It also revealed gains in chromosomes 7 and 19 as well as the loss of chromosomes 10, 12, 13q, and 22q. Radiotherapy with concomitant temozolomide and lomustine was initiated due to young age and promoter methylation [10,11].

## 3. Discussion

The anatomical approach of tractography has been proven to accompany techniques such as transcranial magnetic stimulation or functional MRI in a valuable and reliable fashion [7,12]. However, tractography has decreased accuracy in the cortex, and misinterpretation of false negatives in the precentral gyrus can lead to hemiparesis, while misinterpretation of false positives can lead to an unnecessarily decreased extent of resection [13,14]. The presented case illustrates our approach to steering through this intersection. It is crucial for every patient to be successful in this attempt, since both decreased neurological performance and decreased extent of resection are negative prognostic markers for survival time of patients with glioblastoma [15,16].

Focal epileptic seizures are a common symptom of glioblastoma and require immediate anticonvulsant treatment. Anticonvulsant treatment has a good success rate, which is mirrored by the presented case [10].

Microsurgical tumor resection was assumed to be of high risk in the present case and was therefore meticulously planned as well as thoroughly discussed with the patient. transcranial magnetic stimulation may have yielded valuable information on the exact location of primary motor neurons [17]. However, in our eyes, the risk of further epileptic seizures was too high, so we decided to combine preoperative tractography with intraoperative DES, as described before [18]. Interestingly, the preoperative tractogram suggested that parts of the CST may be found in front of and above the tumor (Figure 1).

Nevertheless, intraoperative DES revealed primary motor neurons to be placed only dorsally to the lesion. Most importantly, DES was in line with the postoperative course of neurological improvement.

This may raise the question of whether DES alone would have been sufficient. We would like to discourage this conclusion, since it is our belief that the correct application of different diagnostic modalities can deliver valuable additional information by complementation. In this case, the success of DES was at least partially due to preoperative tractography, which correctly included primary motor neurons, although it remained partially inconclusive regarding their precise location.

Although fluorescent tissue rims had been left during resection, postoperative MRI disclosed complete resection of the contrast-enhancing tumor parts. Postoperative tractography showed larger portions of the CST dorsally to the situs than preoperatively (Figure 2). One reason for this may be relief from edema and subsequent reestablished white matter integrity of the CST [9]. Another reason may be a false-positive streamline result in preoperative tractography due a diffusion gradient along the tumor capsula, which would mimic the diffusion tensors of nerve fibers. Unfortunately, we cannot further elaborate on this effect. It would even be possible that the preoperative tractogram included premotor connections, which have been severed without postoperative neurological deficit.

Notably, the ventral fibers do not appear severed in postoperative tractography. This may have been important for neurological recovery, as white matter integrity of the premotor regions has been found to be associated with motor output after stroke [19].

As familial glioblastoma is extremely rare in adults, the prior anaplastic ependymoma in the patient’s son was most likely a statistical coincidence [20]. This is especially plausible since histopathological ependymoma is distinct from glioma [8].

## 4. Conclusions

CST tractography can assist in neurosurgical planning for the resection of lesions in the precentral gyrus. In selected cases, the resection of glioblastoma in this region is possible without neurological deficit. However, functional planning is crucial and cannot rely on one technique alone. As each modality has its own limitations, this case highlights the necessity to interpret tractography results with vigilance and consciousness of the underlying physical premises. Patients have to be informed about the high risk of postoperative paresis.

## Data Availability

Data supporting the findings are available from the corresponding author upon reasonable request.
